# Perianal ulcerative skin tuberculosis: A case report

**DOI:** 10.1097/MD.0000000000010836

**Published:** 2018-06-01

**Authors:** Shaofang Wu, Wu Wang, Huan Chen, Wen Xiong, Xin Song, Xinmin Yu

**Affiliations:** a Department of Pathology; b Department of Anesthesiology, Lishui Central Hospital, Lishui, Zhejiang, China.

**Keywords:** pulmonary tuberculosis, skin tuberculosis, ulcer

## Abstract

**Rationale:**

Ulcerative skin tuberculosis (TB) is a rare form of extrapulmonary TB.

**Case report:**

We present a case of a 65-year-old patient with perianal ulcer, which had been present for 1 year. Anamnesis revealed he had been persistently coughing for the same period of time. Histological examination of perianal skin showed necrotizing granulomatous lesions, acid-fast staining in sputum samples was ++++, TB antibody in the blood was positive, TB DNA test was positive, and chest scan that showed secondary pulmonary TB accompanied by possible pulmonary cavity formation in the 2 upper lungs.

**Interventions:**

Anti-TB therapy with isoniazid, rifampicin, ethambutol, and pyrazinamide for 6 months. The skin ulcer completely healed after 6 months.

**Conclusion:**

TB should be suspected for nonhealing ulcers. Pertinent studies should be done early during the lesion; finally, TB treatment should be initiated immediately after diagnosis is made.

## 1 Introduction

Tuberculosis (TB) is one of the oldest human diseases. It is widely spread and a threatening disease in humans. According to World Health Organization, about 20% to 40% of the world's population was affected by *Mycobacterium tuberculosis*, with about 8 to 9 million new cases each year.^[1]^ Of all the TB patients, only 8.4% to 13.7% patients had extrapulmonary manifestations, and only 1% to 1.5% patients with extrapulmonary manifestations had skin TB, with unclearly defined criteria.^[2,3]^ Its clinical manifestations were diverse, hence making it difficult for clinical diagnosis. The most common sites of skin TB are face, neck, and trunk.^[4]^ This study reported a case of perianal ulcerative skin TB.

## 2 Case report

**Figure 1:**
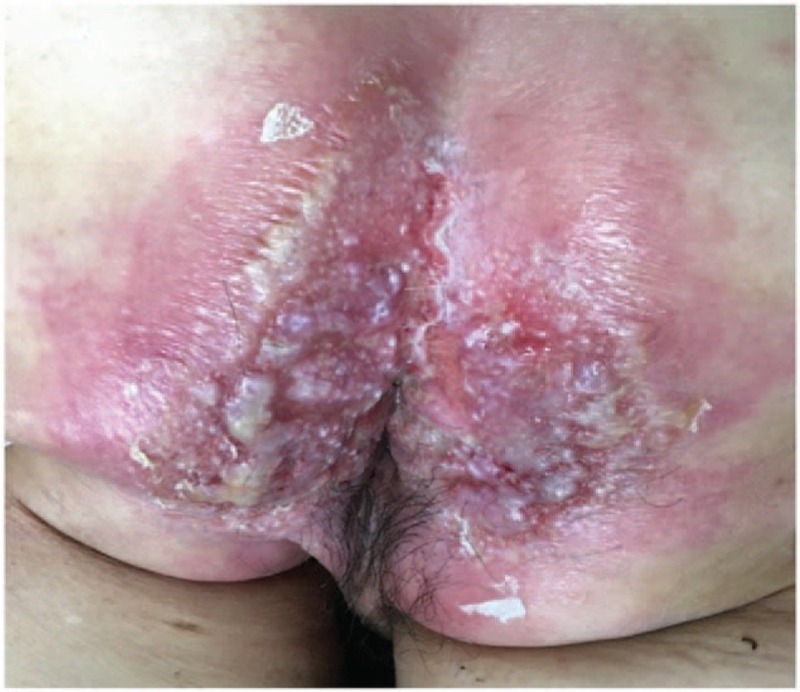
A large erythema around the anus with a size of about 20 cm × 15 cm. Skin ulcers nearly 4 cm range in the perianal area were seen. Fresh granulation, with appearance few purulent secretions on the surface were seen.

**Figure 2:**
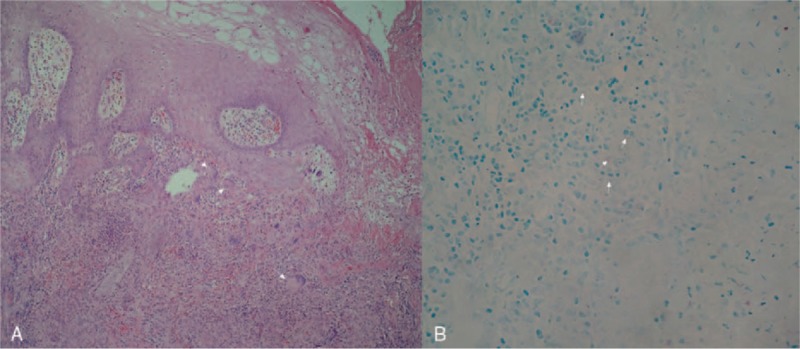
(A) (Perianal skin) necrotic granulomatous lesions (tuberculosis), multinuclear giant cell could be seen (the white arrow showed in the figure, H&E ×400); (B) The typical acid-fast bacilli were found using acid-fast staining (the white arrow shown in the figure, acid-fast staining ×1000).

**Figure 3:**
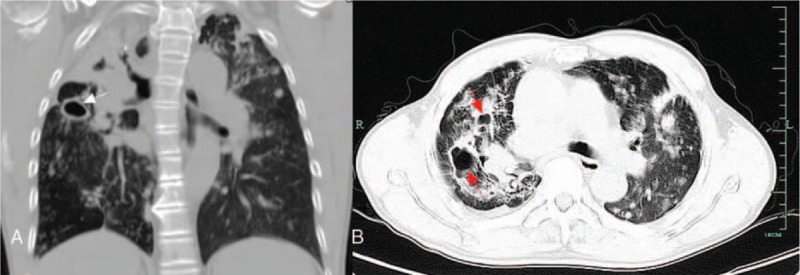
Secondary pulmonary tuberculosis in double lungs with possible cavity formation in the 2 upper lungs, with damaged part of the right upper lung (the white, red arrows in figure showed the cavity).

A 65-year-old male farmer was admitted to the dermatology department of Lishui Central Hospital in April 2016 with the chief complaint of erythema, pruritus, and ulceration of the perianal skin combined with cough, which lasted for 1 year. One year ago, patient had perianal erythema, accompanied by pruritus, ulceration, exudation, and pain. Further questioning revealed that the patient had been coughing several times a day. The patient occasionally had white sputum, without any hemoptysis, chest pain, low grade fever, night sweats, or any other discomfort. The patient had applied a variety of ointments for external use, without improvement. The erythema gradually expanded, affecting half of the hip on both sides of the crissum; an ulcer developed at the center of the erythema. Past medical history included hepatitis B for more than 10 years, and hypertension for about 3 years. The patient had surgical history of cholecystectomy at 39 years of age and denied previous history of TB, tumor, being engaged in risky sexual behaviors, or similar family history. Physical examinations included body temperature of 36.9°C, blood pressure 133/86 mm Hg, pulse rate 86 beats/min, breathing 20 times/min, double pulmonary breath sounded rough without obvious rales. Physical examination by specialist showed a large erythematous plaque of about 20 cm × 15 cm around the anus, skin ulcers could be seen nearly 4 cm range at the perianal area, and the base could be seen with fresh granulation, and few purulent secretions (See Fig. 1). Blood routine test, liver and kidney function tests, treponema pallidum particle agglutination assay (TPPA), toluidine red unheated serum test (TRUST), combined detection of human immunodeficiency virus (HIV) antibodies, and HIV antigens were all negative or within normal ranges. The detection and screening of alpha-fetoprotein (AFP) tumor marker, carcinoembryonic antigen (CEA), squamous cell carcinoma antigen (SCC), total prostate specific antigen (TPSA), and free prostate-specific antigen (FPSA) were all normal. Blood sedimentation rate was 50.0 mm/h, TB DNA was positive, and TB antibody was positive. Acid-fast bacillus detection in sputum samples was ++++. Histological examination of perianal skin showed an ulcerative and necrotic area in the perianal skin, and peripheral epidermis had keratosis and hyperkeratosis. The stratum spinosum was proliferated, accompanied by intercellular edema. The whole dermis had epithelial-like cell mass, Langerhans giant cells could be seen, a large number of infiltrating lymphocytes were observed, and anti-acid staining was positive. Pathological diagnosis demonstrated (perianal skin) necrotic granulomatous lesions (TB). Special staining demonstrated acid-fast bacilli using acid-fast staining, periodic acid-Schiff stain (PAS) was negative (−), silver hexosamine stain was negative (−) (See Fig. 2). Chest computed tomography (CT) scan showed symmetrical thorax, trachea moved to the right, and double lung marking increased and disordered. Diffuse nodular, flocculent, and striped high-density shadow could be seen in both the lungs, the edges were blurred, and the density was uneven. The partial lung tissues in both the upper lungs were consolidated and the cavity was formed. Hilus of the lung and mediastinal lymph nodes were not enlarged. The shape of the heart was not abnormal. There was no pleural effusion in the bilateral pleural cavity. Intrahepatic bile duct showed dilatation. Chest CT scan showed secondary pulmonary TB with cavitation in both upper lobes, and part of the right upper lung was damaged (See Fig. 3). The results of abdominal enhanced CT showed intrahepatic bile duct dilatation and pneumobilia, gallbladder was not shown; splenomegaly, and multiple renal cysts in both the kidneys; possibility of duodenal descending diverticulum, and a little pneumatosis as a partial small intestinal obstruction. Admitting diagnosis showed perianal ulcerative skin TB, secondary bilateral pulmonary TB with possible cavity formation in the 2 upper lungs, TB bacillus was positive (+) in sputum smear. The patient was transferred to the infectious disease department for treatment, and was given with regular anti-TB treatment, which included isoniazid tablets 0.1 g/time, 3 times/day; rifampicin capsules 0.45 g/time, 1 time/day; ethambutol tablets 0.75 g/time, 1 time/day; pyrazinamide tablets 0.75 g/time, 2 times/day. The patient was discharged after 8 days of hospital treatment. *M. tuberculosis* in sputum smear was negative (−) at the time of discharge. Flushing and exudation of perianal skin were better than before. The patient was recommended to take regular anti-TB drugs for 6 months after discharge. After 6 months of discharge, the patient was followed up through telephone and replied that the ulcer had healed.

## 3 Discussion

Extrapulmonary TB accounts for 8.4% to 13.7% of all TB cases; of these, skin TB accounts for only 1% to 1.5%.^[2,3]^ Ulcerative skin TB is also known as TB cutis orificialis, or ulcerative miliary TB. The characteristic skin lesions of this disease mostly occurred in the oral cavity, genital and anal mucosa, and is extremely rare clinically.^[5,6]^ It is believed that occurrence of ulcerative skin TB is caused by hematogenous spread of a preexisting TB infection in another organ or tissue. Mycobacteria is released into the blood and lymph circulation. Infection occurs when immunity of the patient is decreased (or) *M. tuberculosis* is directly implanted into the mucosa further forming an ulcer when further infected with enteric bacteria.^[7]^ Granular nodules appeared on the initial onset of this disease, which were gradually increased and broke down to form small groups of ulcers, and merged with each other forming large ulcers. Pale granulation tissue was present at the base of the ulcer, and the edge was, surrounded by flush, purulent secretions, and mossy membrane. The diagnosis mainly depended on the following aspects: clinical manifestations; skin PPD test; smearing and culture of *M. tuberculosis*; histopathological biopsy; history of TB in other systems or organs; PCR method. Histopathological biopsy remains to be the gold standard for diagnosing TB. However, the examination period was relatively long, which limited its clinical application. Yeboah-Manu et al^[8]^ showed that the comprehensive application of PCR technique helps in diagnosing skin TB with higher efficiency, and the diagnostic sensitivity and specificity were higher than other traditional acid-fast staining smear tests. Rapid and accurate diagnosis of skin TB can be done by PCR utilizing chip diagnostic technology.^[9,10]^ Thus, the experimental diagnosis of skin TB has been gradually developed, from the utilization of traditional laboratory diagnostic methods to currently combining traditional methods with molecular biological techniques for their higher diagnostic efficiency.

## 4 Conclusion

In this case, failure to diagnose pulmonary and cutaneous TB resulted in locally aggressive chronic cutaneous involvement. Further workup should be performed in patients with chronic cough and in chronic nonhealing ulcers to provide expedited TB treatment. Efforts regarding patient education and counseling should be addressed to prevent disability in at-risk populations around the world.

## Acknowledgment

The publication of this study had obtained the patient's consent, and we thanked for the patient's cooperation during the treatment process.

## Author contributions

**Conceptualization:** Shaofang Wu, Wu Wang.

**Data curation:** Shaofang Wu, Wu Wang, Huan Chen, Wen Xiong.

**Formal analysis:** Shaofang Wu, Huan Chen, Xin Song.

**Investigation:** Shaofang Wu, Wu Wang, Huan Chen, Xin Song, Xinmin Yu.

**Project administration:** Xinmin Yu.

**Resources:** Wen Xiong.

**Validation:** Wu Wang.

**Visualization:** Xin Song.

**Writing – original draft:** Shaofang Wu.

**Writing – review & editing:** Wu Wang, Huan Chen, Wen Xiong, Xin Song, Xinmin Yu.

## References

[1] Ugarte-Gil C Ponce M Zamudio C Knowledge about HIV prevention and transmission among recently diagnosed tuberculosis patients: a cross sectional study. BMC Public Health 2013;13:1237. 24373517 10.1186/1471-2458-13-1237 PMC3883486

[2] Ghosh S Aggarwal K Jain VK Tuberculosis verrucosa cutis presenting as diffuse plantar keratoderma: an unusual sight. Indian J Dermatol 2014;59:80–1. 24470667 10.4103/0019-5154.123511 PMC3884935

[3] Sankar MM Singh J Diana SC Molecular characterization of Mycobacterium tuberculosis isolates from North Indian patients with extrapulmonary tuberculosis. Tuberculosis (Edinb) 2013;93:75–83. 23140853 10.1016/j.tube.2012.10.005

[4] Hernandez Solis A Herrera Gonzalez NE Cazarez F Skin biopsy: a pillar in the identification of cutaneous Mycobacterium tuberculosis infection. J Infect Dev Ctries 2012;6:626–31. 22910569 10.3855/jidc.2729

[5] Brown-Gallardo BM Managing cutaneous tuberculosis: a case report. Ostomy Wound Manage 2017;63:34–41. 28759425

[6] Medina-Murillo GR Rodriguez-Medina U Rodriguez-Wong U Perianal cutaneous tuberculosis. Rev Gastroenterol Mex 2017;82:259–60. 28372845 10.1016/j.rgmx.2017.02.002

[7] Bravo FG Gotuzzo E Cutaneous tuberculosis. Clin Dermatol 2007;25:173–80. 17350496 10.1016/j.clindermatol.2006.05.005

[8] Yeboah-Manu D Asante-Poku A Asan-Ampah K Combining PCR with microscopy to reduce costs of laboratory diagnosis of Buruli ulcer. Am J Trop Med Hyg 2011;85:900–4. 22049046 10.4269/ajtmh.2011.11-0362 PMC3205638

[9] Guo Y Zhou Y Wang C Rapid, accurate determination of multidrug resistance in M. tuberculosis isolates and sputum using a biochip system. Int J Tuberc Lung Dis 2009;13:914–20. 19555544

[10] Gordon SV Brosch R Billault A Identification of variable regions in the genomes of tubercle bacilli using bacterial artificial chromosome arrays. Mol Microbiol 1999;32:643–55. 10320585 10.1046/j.1365-2958.1999.01383.x

[11] Das CK Mahapatra A Das MM Coexistence of cutaneous tuberculosis (scrofuloderma) and hanseniasis: a rare presentation. J Clin Diagn Res 2014;8:141–2. 24701508 10.7860/JCDR/2014/7050.4033 PMC3972534

[12] Wang H Wu Q Lin L Cutaneous tuberculosis: a diagnostic and therapeutic study of 20 cases. J Dermatolog Treat 2011;22:310–4. 20673149 10.3109/09546634.2010.487889

